# Untargeted lipidomics reveals racial differences in lipid species among women

**DOI:** 10.1186/s40364-024-00635-4

**Published:** 2024-08-09

**Authors:** Ghazaleh Pourali, Liang Li, Kayla R. Getz, Myung Sik Jeon, Jingqin Luo, Chongliang Luo, Adetunji T. Toriola

**Affiliations:** 1 Department of Surgery, Division of Public Health Sciences, Washington University School of Medicine, 660 South Euclid Avenue, Campus, Box 8100, St. Louis, MO 63110 USA; 2 Institute for Informatics, Data Science & Biostatistics, School of Medicine, Washington University, St. Louis, MO USA; 3 Siteman Cancer Center Biostatistics Shared Resource, Division of Public Health Sciences, Department of Surgery, Washington University School of Medicine, St. Louis, MO USA; 4 Siteman Cancer Center, Washington University School of Medicine, St. Louis, MO USA

**Keywords:** Lipidomics, Lipids, Non-Hispanic Black, Non-Hispanic White, Body Mass Index, Women, Triacylglycerols

## Abstract

Understanding the biological mechanisms underlying racial differences in diseases is crucial to developing targeted prevention and treatment. There is, however, limited knowledge of the impact of race on lipids. To address this, we performed comprehensive lipidomics analyses to evaluate racial differences in lipid species among 506 non-Hispanic White (NHW) and 163 non-Hispanic Black (NHB) women. Plasma lipidomic profiling quantified 982 lipid species. We used multivariable linear regression models, adjusted for confounders, to identify racial differences in lipid species and corrected for multiple testing using a Bonferroni-adjusted p-value < 10^–5^. We identified 248 lipid species that were significantly associated with race. NHB women had lower levels of several lipid species, most notably in the triacylglycerols sub-pathway (*N* = 198 out of 518) with 46 lipid species exhibiting an absolute percentage difference ≥ 50% lower in NHB compared with NHW women. We report several novel differences in lipid species between NHW and NHB women, which may underlie racial differences in health and have implications for disease prevention.

**Supplementary Information:**

The online version contains supplementary material available at 10.1186/s40364-024-00635-4.

To the editor,

Non-Hispanic Black populations (NHBs) experience higher rates of chronic diseases, including metabolic disorders compared with non-Hispanic White populations (NHWs) [1]. While social determinants of health contribute to these differences, evaluating possible biological contributors to the observed racial differences is crucial to designing effective health interventions. Lipids play key roles in cellular functions and diseases [2]. Due to a lack of data, we performed the first comprehensive analysis of lipidomic profiles in NHB and NHW women to determine whether racial differences exist. This is critical to understanding the biological basis of racial disparities in disease incidence and outcomes.

Our study included 669 women [506 NHW (75.6%) and 163 NHB (24.4%)] recruited at Washington University School of Medicine, St. Louis, MO. A detailed description of this study population has been published [3]. Our lipidomic profiling performed at Metabolon identified 982 lipid species within 3 super-pathways and 14 sub-pathways. We excluded 125 out of 982 lipid species missing in ≥ 300 of the women for the analyses. We investigated the associations of lipid species with race using linear regression models, adjusting for confounders, and accounted for multiple testings by applying the Bonferroni correction. The study methods are detailed in the Supplementary Methods. Participants' characteristics are described in Table S1. NHB women had higher BMI (34.6 kg/m^2^) compared with NHW women (28.7 kg/m^2^) (*p*-value < 0.001). Additionally, a higher proportion of NHB women reported no alcohol consumption (52.8%) compared to NHW women (21.5%) (*p*-value < 0.001).

We observed significant racial differences at the super-pathway, sub-pathway, and species levels in 248 lipid species out of the 857 lipid species (28.9%) (Bonferroni-adjusted p-value < 10^–5^) (Fig. 1, Table S2). These species belong to triacylglycerols (TAG, *n* = 198), diacylglycerols (DAG, *n* = 19), phosphatidylcholines (PC, *n* = 14), phosphatidylethanolamines (PE, *n* = 4), cholesteryl esters (CE, *n* = 3), lysophosphatidylcholines (LPC, *n* = 3), lysophosphatidylethanolamines (LPE, *n* = 3), sphingomyelins (SM, *n* = 2), ceramides (CER, *n* = 1), and phosphatidylinositols (PI, *n* = 1) sub-pathways. All lipid species except TAG58:10-FA20:4 were lower in NHB women. Forty-six lipid species [TAG (*n* = 45) and DAG (*n* = 1)] exhibited an absolute percentage difference ≥ 50% (Table 1). The top 5 species with the largest absolute percentage differences were all lower in NHB than in NHW: TAG46:2-FA16:1 (60.9%, *p* = 2.8 × 10^–12^), TAG44:0-FA14:0 (59.8%, *p* = 9.5 × 10^–6^), TAG47:2-FA16:1 (59.8%, *p* = 4.6 × 10^–16^), TAG46:1-FA16:1 (59.3%, *p* = 1.9 × 10^–9^), and TAG44:1-FA14:0 (58.8%, *p* = 4.8 × 10^–6^). At absolute percentage difference thresholds of ≥ 40%, ≥ 30%, and ≥ 20%, we identified 120, 201, and 238 lipid species, respectively, that were significantly associated with race. (Table S3). At the lipid sub-pathway level, 4 out of 14 lipid sub-pathways including TAG, DAG, LPE, and PC, were significantly lower in NHB women compared with NHW women (Table S4). TAG sub-pathway showed significant enrichment (*p*-value of 2.1 × 10^–14^), with 198 out of the 518 total species in this sub-pathway significantly different between NHB and NHW women (Table S5).

**Fig. 1:**
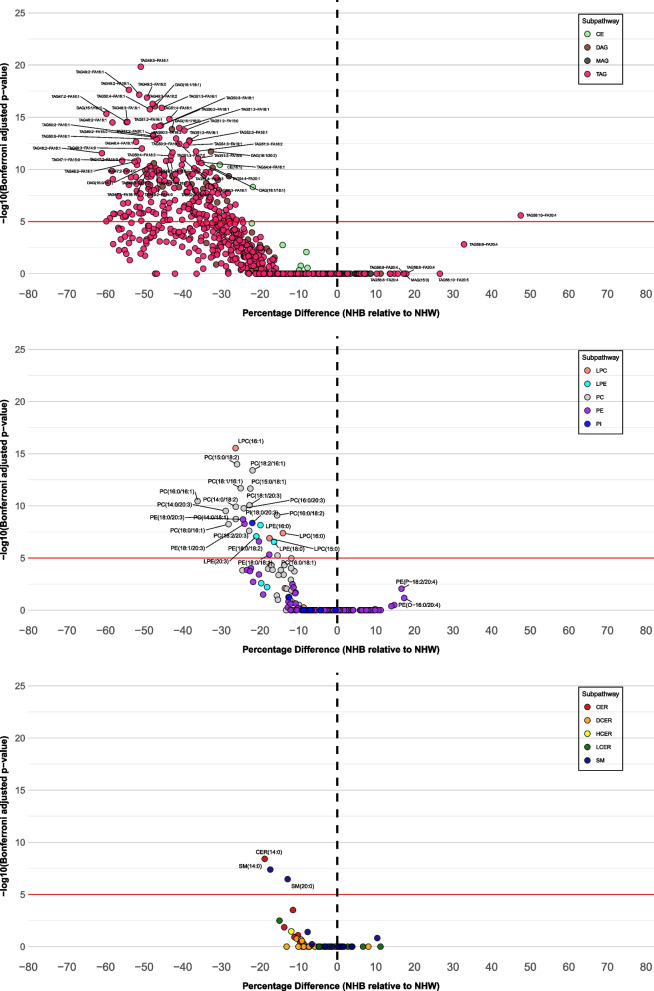
Associations of Race with Lipid Species by Super-pathway. Multivariable linear regression analysis between the log-transformed lipid species and race (non-Hispanic Black, non-Hispanic White) was performed, adjusted for age, BMI at enrollment, alcohol consumption, and education. Multiple hypothesis testing was corrected using Bonferroni correction. Percentage differences were back-transformed linear regression coefficients, calculated as ${100\times (e}^{\beta }-1)$, with a 95% confidence interval. Volcano plots of log Bonferroni-adjusted *p*-values were generated against the percentage differences. **A** Associations of race with lipid species in neutral complex lipids super-pathway. Names of neutral complex lipid species with Bonferroni-adjusted p-values <${10}^{-10}$ or percentage differences > 15% were labeled. **B** Associations of race with lipid species in phospholipid super-pathway. Names of phospholipid species with Bonferroni-adjusted *p*-values <${10}^{-5}$ or percentage differences > 15% were labeled. **C** Associations of race with lipid species in sphingolipid super-pathway. Names of sphingolipid species with Bonferroni-adjusted *p*-values <${10}^{-5}$ or percentage differences > 15% were labeled. BMI, body mass index; NHB, non-Hispanic Black; NHW, non-Hispanic White; CE, cholesteryl ester; CER, ceramide; DAG, diacylglycerol; DCER, dihydroceramide; HCER, hexosylceramide; LCER, lactosylceramide; LPC, lysophosphatidylcholine; LPE, lysophosphatidylethanolamine; MAG, monoacylglycerol; PC, phosphatidylcholine; PE, phosphatidylethanolamine; PI, phosphatidylinositol; SM, sphingomyelin; TAG, triacylglycerol

**Table 1:** Lipid Species with ≥ 50% Absolute Percentage Difference between non-Hispanic Black and non-Hispanic White Women^a,b^

Lipid Species	Percentage Difference (%)^c^	Bonferroni Adjusted *P*-value
TAG46:2-FA16:1	-60.9 (-68.9, -50.9)	2.8E-12
TAG44:0-FA14:0	-59.8 (-70.5, -45.2)	9.5E-06
TAG47:2-FA16:1	-59.8 (-66.9, -51.1)	4.6E-16
TAG46:1-FA16:1	-59.3 (-68.2, -48.0)	1.9E-09
TAG44:1-FA14:0	-58.8 (-69.3, -44.7)	4.8E-06
TAG46:2-FA14:1	-58.4 (-66.8, -47.7)	1.4E-10
TAG48:2-FA16:1	-58.2 (-65.5, -49.4)	3.2E-15
TAG46:1-FA14:0	-58.1 (-66.9, -47.0)	8.5E-10
TAG44:1-FA16:1	-58.0 (-68.2, -44.4)	1.8E-06
TAG44:1-FA14:1	-57.6 (-67.9, -44.0)	1.9E-06
TAG44:2-FA16:1	-56.6 (-66.0, -44.6)	3.8E-08
TAG45:0-FA15:0	-56.1 (-66.4, -42.8)	1.6E-06
TAG47:1-FA15:0	-55.7 (-63.9, -45.7)	1.5E-11
TAG45:0-FA14:0	-55.5 (-65.9, -41.9)	3.8E-06
TAG46:1-FA16:0	-55.4 (-65.2, -43.0)	1.9E-07
TAG46:0-FA14:0	-54.9 (-65.3, -41.5)	2.8E-06
TAG46:0-FA16:0	-54.8 (-65.1, -41.3)	3.4E-06
TAG47:1-FA14:0	-54.6 (-63.1, -44.3)	1.5E-10
DAG(16:1/16:1)	-54.6 (-61.8, -46.0)	3.0E-15
TAG48:3-FA16:1	-54.3 (-61.5, -45.8)	2.8E-15
TAG45:1-FA15:0	-54.2 (-63.8, -42.0)	1.6E-07
TAG45:0-FA16:0	-54.0 (-64.1, -41.0)	1.2E-06
TAG49:2-FA16:1	-54.0 (-60.6, -46.2)	2.4E-18
TAG46:2-FA14:0	-53.9 (-62.8, -42.9)	3.0E-09
TAG47:1-FA16:0	-53.4 (-62.0, -42.8)	5.7E-10
TAG46:1-FA14:1	-53.1 (-63.3, -40.0)	2.1E-06
TAG48:2-FA18:1	-52.5 (-60.6, -42.7)	1.8E-11
TAG47:1-FA18:1	-52.1 (-60.7, -41.7)	5.2E-10
TAG48:4-FA16:1	-52.1 (-59.6, -43.1)	2.3E-13
TAG46:3-FA14:1	-52.0 (-60.8, -41.2)	2.4E-09
TAG46:3-FA12:0	-52.0 (-61.8, -39.7)	3.9E-07
TAG46:3-FA14:0	-51.9 (-60.9, -40.7)	1.1E-08
TAG47:2-FA14:0	-51.9 (-60.0, -42.0)	3.7E-11
TAG48:1-FA16:1	-51.6 (-60.7, -40.3)	1.9E-08
TAG47:2-FA15:0	-51.6 (-59.6, -42.0)	1.4E-11
TAG47:0-FA14:0	-51.3 (-61.4, -38.6)	1.6E-06
TAG49:2-FA18:1	-51.3 (-57.9, -43.6)	6.9E-18
TAG47:0-FA15:0	-51.1 (-60.9, -39.0)	4.2E-07
TAG46:2-FA18:1	-50.9 (-60.3, -39.5)	5.5E-08
TAG46:3-FA16:1	-50.9 (-60.0, -39.7)	2.1E-08
TAG49:3-FA16:1	-50.9 (-57.0, -43.8)	1.5E-20
TAG48:1-FA18:0	-50.8 (-60.8, -38.1)	1.7E-06
TAG46:1-FA18:1	-50.8 (-61.2, -37.4)	8.4E-06
TAG48:1-FA16:0	-50.7 (-59.4, -40.2)	1.7E-09
TAG48:3-FA14:0	-50.6 (-58.2, -41.5)	1.0E-12
TAG47:0-FA16:0	-50.3 (-60.3, -37.8)	1.5E-06

^a^Multivariable linear regression analysis was performed on log-transformed lipid species and adjusted for age, body mass index (BMI), alcohol consumption, and education

^b^Multiple hypothesis testing was corrected using Bonferroni correction. Statistical significance was defined as a Bonferroni-adjusted *p*-value < ${10}^{-5}$

^c^Percentage difference was back-transformed linear regression coefficients, calculated as $100\times ({e}^{\beta }-1)$, with a 95% confidence interval. A negative percentage difference indicates that the lipid species levels are lower in non-Hispanic black women compared with non-Hispanic white women

This is the first study to comprehensively use untargeted lipidomic profiling to identify differences in lipid species between NHW and NHB women. Other studies that have compared lipid levels in NHB individuals with NHW individuals analyzed only a limited number of lipids, such as cholesterol, LDL, and HDL [4, 5]. Our approach reveals novel insights that have not been captured in prior studies. A recent study of 175 women and 175 men using a metabolomics approach identified racial differences in some lipids [6]. Our study population of 669 women is much larger and we analyzed a broader range of lipid species which provides greater power to detect smaller differences. This broader coverage, particularly in TAGs, allows for a more comprehensive assessment of racial differences in lipid species and pathways.

The only lipid species higher in NHB women was TAG58:10-FA20:4. Our finding is similar to a prior report of higher levels of long-chain polyunsaturated TAGs (C56-C60 with 8–12 double-bonds) in NHBs [7]. Similar to the report by Cai et al. [8], our data confirms lower levels of PC(16:0/18:1) in NHB women. Unlike their study, which employed pooled samples from only 35 individuals and targeted 193 lipids, we analyzed individual samples using Metabolon's untargeted approach along with robust data analysis and adjustment for confounders. This comprehensive approach minimizes bias due to sample pooling and allows for a robust evaluation of racial differences in lipids. Prior research associates low levels of specific PC species, including PC(33:1)[PC(15:0/18:1)] and PC(33:2)[PC(15:0/18:2)], with increased risk of coronary artery disease and myocardial infarction [9]. Notably, these same PC species were found to be significantly lower in NHB women in our study, which warrants further investigation to determine if these reduced PC levels contribute to health disparities in NHB women.

Our findings highlight the importance of evaluating racial differences in molecular markers relevant to health and can inform targeted interventions and personalized disease prevention. Future studies should confirm whether these differences contribute to racial health disparities and disease susceptibility.

### Supplementary Information

Supplementary Material 1

## Data Availability

Data described in the manuscript, code book, and analytic code will be made available upon request and approval.

## Abbreviations

NHW: Non-Hispanic White
NHB: Non-Hispanic Black
CE: Cholesteryl esters
DAG: Diacylglycerols
TAG: Triacylglycerols
MAG: Monoacylglycerols
PC: Phosphatidylcholines
PI: Phosphatidylinositols
PE: Phosphatidylethanolamines
LPC: Lysophosphatidylcholines
LPE: Lysophosphatidylethanolamines
CER: Ceramides
DCER: Dihydroceramides
HCER: Hexosylceramides
LCER: Lactosylceramides
SM: Sphingomyelins
