# Supplementary material for: Untargeted lipidomics reveals racial differences in lipid species among women
Source: Biomark Res. 2024 Aug 9;12:79. doi: 10.1186/s40364-024-00635-4 (PMC11312829; doi:10.1186/s40364-024-00635-4)
Supplement: Supplementary file 1 — Supplementary Material 1 [file 40364_2024_635_MOESM1_ESM.docx]

**Supplementary Methods**

**Study Population**

Our analysis was performed on 669 NHB and NHW women who were recruited while undergoing annual screening mammograms at Washington University (WU) School of Medicine, St. Louis, MO. Women were enrolled if they were not pregnant and had no history of breast augmentation, cancer, or recent use of selective estrogen receptor modulators in the prior six months (1). Study participants provided written informed consent (1). Ethical approval was obtained from the WU Institutional Review Board, adhering to the Declaration of Helsinki. Participants completed a questionnaire detailing demographic, behavioral, reproductive, and clinical information, while weight and height measurements were taken on the day of their mammography visit. Fasting blood samples were collected and stored at -80°C within 30 minutes of collection (2).

**Lipidomic Profiling**

Each sample consisted of 0.25 mL of plasma, which was sent to Metabolon (Durham, NC®) for comprehensive lipidomic profiling using Metabolon's ultra-high performance liquid chromatography-tandem mass spectrometry (UPLC-MS/MS) approach. This analysis identified 982 lipid species within 3 super-pathways and 14 sub-pathways. The lipid super pathways included neutral complex lipids (including cholesteryl esters (CE), diacylglycerols (DAG), triacylglycerols (TAG), and monoacylglycerols (MAG)), phospholipids (including phosphatidylcholines (PC), phosphatidylinositols (PI), phosphatidylethanolamines (PE), lysophosphatidylcholines (LPC), and lysophosphatidylethanolamines (LPE)), and sphingolipids (including ceramides (CER), dihydroceramides (DCER), hexosylceramides (HCER), lactosylceramides (LCER), and sphingomyelins (SM)). To quantify each lipid species, its signal intensity was normalized against a corresponding internal standard. The amount of internal standard added to the sample was then factored in to determine the final concentration (3). Quality control samples, a large pool of human plasma maintained by Metabolon, were also included in the analysis runs to ensure accuracy. The overall variation between replicate analyses was assessed by calculating the median relative standard deviation (RSD), which came out to be 8%. To obtain sub-pathway concentrations, the levels of all individual lipid species within a sub-pathway were summed (3).

**Statistical Analysis**

For the analyses, we used combat normalized values to account for potential batch effects. We excluded 125 out of 982 lipid species with excessive missing values (i.e., missing in ≥300 of the women). For the remaining missing values, we employed the 10-nearest neighbor method imputation. We investigated the associations of lipid species with race using multivariable linear regression models adjusted for age (continuous), BMI (continuous), alcohol consumption (yes, no), and education level (high school or less, post-high school/some college, college graduate, and postgraduate). In these models, variables such as oral contraceptive use (yes, no) and family history of breast cancer (yes, no) were initially considered but ultimately excluded because they had minimal impact on lipid species to be considered as confounders. To satisfy the assumptions of normality and homoscedasticity, we log-transformed the lipid concentrations for the regression analyses. Linear regression coefficients were subsequently back-transformed in the original scale as percentage differences. We accounted for multiple testings by applying the Bonferroni correction to regression analyses. Pathway enrichment analyses were performed for each sub-pathway where the number of differential lipid species assumed a hypergeometric distribution. Statistical significance was defined as a Bonferroni-adjusted p-value <10^-5^.

**Table S1. Characteristics of 669 Women Recruited from Annual Screening Mammogram at the Joanne Knight Breast Health Center, Washington University School of Medicine, St. Louis, MO.**

|  | Overall | Non-Hispanic White | Non-Hispanic Black | P-value |
| --- | --- | --- | --- | --- |
| Number | 669 | 506 | 163 |  |
| Age at Enrollment, Mean (SD) | 46.0 (4.5) | 45.8 (4.6) | 46.5 (4.1) | 0.085 |
| BMI, Mean (SD) | 30.1 (7.4) | 28.7 (6.7) | 34.6 (7.7) | <0.001 |
| Body Fat Percentage, Mean (SD) | 40.7 (11.0) | 38.9 (10.4) | 46.3 (11.1) | <0.001 |
| Family History of Breast Cancer, n (%) | | | | 0.002 |
| No | 516 (77.1) | 384 (75.9) | 132 (81.0) |  |
| Yes | 141 (21.1) | 117 (23.1) | 24 (14.7) |  |
| Missing | 12 (1.8) | 5 (1.0) | 7 (4.3) |  |
| Oral Contraceptive Use, n (%) | | | | 0.002 |
| No | 73 (10.9) | 44 (8.7) | 29 (17.8) |  |
| Yes | 596 (89.1) | 462 (91.3) | 134 (82.2) |  |
| Alcohol Consumption, n (%) | | | | <0.001 |
| No | 195 (29.1) | 109 (21.5) | 86 (52.8) |  |
| Yes | 474 (70.9) | 397 (78.5) | 77 (47.2) |  |
| Education, n (%) |  |  |  | <0.001 |
| High school or less | 50 (7.5) | 18 (3.6) | 32 (19.6) |  |
| Post-high school/some college | 104 (15.5) | 63 (12.5) | 41 (25.2) |  |
| College Graduate | 234 (35.0) | 197 (38.9) | 37 (22.7) |  |
| Postgraduate | 246 (36.8) | 212 (41.9) | 34 (20.9) |  |
| Missing | 35 (5.2) | 16 (3.2) | 19 (11.7) |  |

Abbreviations: standard deviation (SD), body mass index (BMI).

**Table S2. Lipid Species Associated with Race (non-Hispanic Black vs non-Hispanic White) ^a, b^**

| Lipid Species | Percentage Difference (%) ^c^ | Bonferroni Adjusted P-value |
| --- | --- | --- |
| TAG46:2-FA16:1 | -60.9 (-68.9, -50.9) | 2.8E-12 |
| TAG44:0-FA14:0 | -59.8 (-70.5, -45.2) | 9.5E-06 |
| TAG47:2-FA16:1 | -59.8 (-66.9, -51.1) | 4.6E-16 |
| TAG46:1-FA16:1 | -59.3 (-68.2, -48.0) | 1.9E-09 |
| TAG44:1-FA14:0 | -58.8 (-69.3, -44.7) | 4.8E-06 |
| TAG46:2-FA14:1 | -58.4 (-66.8, -47.7) | 1.4E-10 |
| TAG48:2-FA16:1 | -58.2 (-65.5, -49.4) | 3.2E-15 |
| TAG46:1-FA14:0 | -58.1 (-66.9, -47.0) | 8.5E-10 |
| TAG44:1-FA16:1 | -58.0 (-68.2, -44.4) | 1.8E-06 |
| TAG44:1-FA14:1 | -57.6 (-67.9, -44.0) | 1.9E-06 |
| TAG44:2-FA16:1 | -56.6 (-66.0, -44.6) | 3.8E-08 |
| TAG45:0-FA15:0 | -56.1 (-66.4, -42.8) | 1.6E-06 |
| TAG47:1-FA15:0 | -55.7 (-63.9, -45.7) | 1.5E-11 |
| TAG45:0-FA14:0 | -55.5 (-65.9, -41.9) | 3.8E-06 |
| TAG46:1-FA16:0 | -55.4 (-65.2, -43.0) | 1.9E-07 |
| TAG46:0-FA14:0 | -54.9 (-65.3, -41.5) | 2.8E-06 |
| TAG46:0-FA16:0 | -54.8 (-65.1, -41.3) | 3.4E-06 |
| TAG47:1-FA14:0 | -54.6 (-63.1, -44.3) | 1.5E-10 |
| DAG(16:1/16:1) | -54.5 (-61.8, -46.0) | 3.0E-15 |
| TAG48:3-FA16:1 | -54.3 (-61.5, -45.8) | 2.8E-15 |
| TAG45:1-FA15:0 | -54.2 (-63.8, -42.0) | 1.6E-07 |
| TAG45:0-FA16:0 | -54.0 (-64.1, -41.0) | 1.2E-06 |
| TAG49:2-FA16:1 | -54.0 (-60.6, -46.2) | 2.4E-18 |
| TAG46:2-FA14:0 | -53.9 (-62.8, -42.9) | 3.0E-09 |
| TAG47:1-FA16:0 | -53.4 (-62.0, -42.8) | 5.7E-10 |
| TAG46:1-FA14:1 | -53.1 (-63.3, -40.0) | 2.1E-06 |
| TAG48:2-FA18:1 | -52.5 (-60.6, -42.7) | 1.8E-11 |
| TAG47:1-FA18:1 | -52.1 (-60.7, -41.7) | 5.2E-10 |
| TAG48:4-FA16:1 | -52.1 (-59.6, -43.1) | 2.3E-13 |
| TAG46:3-FA14:1 | -52.0 (-60.8, -41.2) | 2.4E-09 |
| TAG46:3-FA12:0 | -52.0 (-61.8, -39.7) | 3.9E-07 |
| TAG46:3-FA14:0 | -51.9 (-60.9, -40.7) | 1.1E-08 |
| TAG47:2-FA14:0 | -51.9 (-60.0, -42.0) | 3.7E-11 |
| TAG48:1-FA16:1 | -51.6 (-60.7, -40.3) | 1.9E-08 |
| TAG47:2-FA15:0 | -51.6 (-59.6, -42.0) | 1.4E-11 |
| TAG47:0-FA14:0 | -51.3 (-61.4, -38.6) | 1.6E-06 |
| TAG49:2-FA18:1 | -51.3 (-57.9, -43.6) | 6.9E-18 |
| TAG47:0-FA15:0 | -51.1 (-60.9, -39.0) | 4.2E-07 |
| TAG46:2-FA18:1 | -50.9 (-60.3, -39.5) | 5.5E-08 |
| TAG46:3-FA16:1 | -50.9 (-60.0, -39.7) | 2.1E-08 |
| TAG49:3-FA16:1 | -50.9 (-57.0, -43.8) | 1.5E-20 |
| TAG48:1-FA18:0 | -50.8 (-60.8, -38.1) | 1.7E-06 |
| TAG46:1-FA18:1 | -50.7 (-61.2, -37.4) | 8.4E-06 |
| TAG48:1-FA16:0 | -50.7 (-59.4, -40.2) | 1.7E-09 |
| TAG48:3-FA14:0 | -50.6 (-58.2, -41.5) | 1.0E-12 |
| TAG47:0-FA16:0 | -50.3 (-60.3, -37.8) | 1.5E-06 |
| TAG48:2-FA16:0 | -49.7 (-57.9, -39.9) | 1.3E-10 |
| TAG48:2-FA14:1 | -49.3 (-57.7, -39.4) | 2.7E-10 |
| TAG49:3-FA15:0 | -49.3 (-55.8, -41.7) | 1.3E-17 |
| TAG48:3-FA18:2 | -49.1 (-57.3, -39.4) | 9.8E-11 |
| TAG49:1-FA16:1 | -49.1 (-57.5, -39.0) | 5.3E-10 |
| TAG48:1-FA18:1 | -48.9 (-57.6, -38.6) | 2.5E-09 |
| TAG48:4-FA18:3 | -48.9 (-57.7, -38.2) | 7.2E-09 |
| TAG48:0-FA14:0 | -48.8 (-58.6, -36.8) | 7.9E-07 |
| TAG48:1-FA14:0 | -48.8 (-57.5, -38.2) | 4.4E-09 |
| TAG50:4-FA16:1 | -48.5 (-55.2, -40.8) | 1.7E-16 |
| TAG47:1-FA16:1 | -48.5 (-56.5, -39.0) | 4.8E-11 |
| TAG49:1-FA17:0 | -48.4 (-57.1, -38.1) | 3.1E-09 |
| TAG50:1-FA20:1 | -48.4 (-57.5, -37.5) | 3.1E-08 |
| TAG49:1-FA14:0 | -48.2 (-57.1, -37.5) | 1.3E-08 |
| TAG48:1-FA14:1 | -48.2 (-57.5, -36.8) | 1.4E-07 |
| TAG48:3-FA16:0 | -48.2 (-56.5, -38.3) | 4.8E-10 |
| TAG48:2-FA14:0 | -47.9 (-56.1, -38.1) | 2.9E-10 |
| TAG49:3-FA18:2 | -47.8 (-54.4, -40.3) | 5.2E-17 |
| TAG45:1-FA18:1 | -47.7 (-57.5, -35.7) | 1.2E-06 |
| TAG50:2-FA16:1 | -47.6 (-54.9, -39.3) | 6.4E-14 |
| TAG52:2-FA20:1 | -47.6 (-54.8, -39.3) | 5.1E-14 |
| DAG(16:0/16:1) | -47.5 (-55.4, -38.2) | 2.6E-11 |
| TAG47:2-FA18:2 | -47.4 (-56.2, -36.9) | 9.7E-09 |
| TAG47:2-FA18:1 | -47.3 (-55.4, -37.8) | 1.2E-10 |
| TAG51:2-FA16:1 | -47.3 (-54.3, -39.2) | 8.0E-15 |
| DAG(16:1/18:1) | -47.2 (-53.8, -39.6) | 9.1E-17 |
| TAG46:3-FA18:2 | -46.9 (-56.8, -34.9) | 1.8E-06 |
| TAG49:2-FA14:0 | -46.9 (-54.9, -37.5) | 8.1E-11 |
| TAG50:5-FA16:1 | -46.8 (-54.0, -38.4) | 1.3E-13 |
| TAG50:1-FA16:1 | -46.6 (-54.7, -37.2) | 1.5E-10 |
| TAG48:4-FA14:0 | -46.6 (-55.2, -36.3) | 6.7E-09 |
| TAG48:3-FA14:1 | -46.2 (-54.3, -36.7) | 2.5E-10 |
| TAG48:4-FA14:1 | -46.1 (-54.1, -36.7) | 1.2E-10 |
| TAG49:2-FA15:0 | -46.1 (-53.2, -37.9) | 9.4E-14 |
| TAG49:2-FA17:0 | -46.1 (-54.4, -36.3) | 1.1E-09 |
| TAG50:3-FA16:1 | -46.0 (-52.9, -38.1) | 6.9E-15 |
| TAG50:2-FA18:1 | -45.6 (-52.5, -37.8) | 6.2E-15 |
| TAG51:3-FA16:1 | -45.5 (-52.0, -38.1) | 1.3E-16 |
| TAG45:1-FA16:0 | -45.2 (-54.5, -34.0) | 3.3E-07 |
| DAG(14:0/16:1) | -45.2 (-53.7, -35.2) | 4.4E-09 |
| TAG49:0-FA18:0 | -45.1 (-55.1, -32.7) | 8.5E-06 |
| TAG49:0-FA15:0 | -45.0 (-54.0, -34.1) | 1.3E-07 |
| TAG49:1-FA15:0 | -44.9 (-52.9, -35.5) | 2.7E-10 |
| TAG50:2-FA20:2 | -44.8 (-54.5, -33.0) | 2.4E-06 |
| TAG48:3-FA18:3 | -44.8 (-54.3, -33.2) | 1.3E-06 |
| TAG49:1-FA18:1 | -44.8 (-52.8, -35.3) | 3.8E-10 |
| TAG50:1-FA18:0 | -44.7 (-53.4, -34.4) | 2.1E-08 |
| TAG49:1-FA16:0 | -44.7 (-52.9, -35.0) | 1.3E-09 |
| TAG49:2-FA16:0 | -44.3 (-51.9, -35.5) | 1.6E-11 |
| TAG49:0-FA16:0 | -43.9 (-53.8, -31.9) | 7.1E-06 |
| TAG50:4-FA20:3 | -43.7 (-52.9, -32.6) | 5.3E-07 |
| TAG52:8-FA16:1 | -43.6 (-51.8, -34.0) | 2.0E-09 |
| TAG51:4-FA16:1 | -43.5 (-50.0, -36.0) | 1.5E-15 |
| TAG48:2-FA18:2 | -43.4 (-52.4, -32.8) | 1.6E-07 |
| TAG48:3-FA18:1 | -43.3 (-53.1, -31.4) | 6.7E-06 |
| TAG49:3-FA16:0 | -43.2 (-50.7, -34.6) | 1.3E-11 |
| TAG50:4-FA18:3 | -43.0 (-50.3, -34.6) | 5.1E-12 |
| DAG(16:1/18:2) | -42.8 (-49.5, -35.2) | 1.4E-14 |
| DAG(14:0/18:1) | -42.7 (-50.6, -33.5) | 4.4E-10 |
| TAG50:3-FA16:0 | -42.7 (-49.9, -34.4) | 2.4E-12 |
| TAG49:3-FA18:3 | -42.2 (-50.4, -32.6) | 6.0E-09 |
| TAG50:1-FA14:0 | -42.0 (-50.7, -31.8) | 9.1E-08 |
| TAG52:1-FA20:0 | -42.0 (-49.7, -33.0) | 3.0E-10 |
| TAG52:2-FA14:0 | -41.8 (-49.7, -32.5) | 1.3E-09 |
| TAG50:3-FA18:2 | -41.7 (-48.5, -34.0) | 1.1E-13 |
| TAG49:2-FA18:2 | -41.6 (-49.5, -32.5) | 1.1E-09 |
| DAG(14:0/18:2) | -40.9 (-49.0, -31.6) | 5.2E-09 |
| TAG51:2-FA18:1 | -40.9 (-47.5, -33.6) | 1.1E-14 |
| TAG47:1-FA17:0 | -40.9 (-50.0, -30.1) | 9.9E-07 |
| TAG51:3-FA17:0 | -40.9 (-47.8, -33.1) | 3.6E-13 |
| TAG50:2-FA14:0 | -40.8 (-48.3, -32.1) | 1.5E-10 |
| TAG52:3-FA20:2 | -40.3 (-47.6, -31.9) | 3.7E-11 |
| TAG54:7-FA22:6 | -40.2 (-49.6, -28.9) | 6.8E-06 |
| TAG50:3-FA18:1 | -40.0 (-47.1, -31.8) | 1.0E-11 |
| TAG50:2-FA18:0 | -39.7 (-48.5, -29.5) | 4.3E-07 |
| TAG51:2-FA15:0 | -39.6 (-46.0, -32.3) | 1.9E-14 |
| TAG51:3-FA16:0 | -39.5 (-48.2, -29.4) | 3.0E-07 |
| TAG54:3-FA16:1 | -39.1 (-45.8, -31.5) | 4.9E-13 |
| TAG50:4-FA16:0 | -39.0 (-47.1, -29.8) | 1.4E-08 |
| TAG51:1-FA15:0 | -38.9 (-46.6, -30.0) | 2.6E-09 |
| TAG52:3-FA14:0 | -38.8 (-46.8, -29.6) | 1.4E-08 |
| TAG51:1-FA18:0 | -38.6 (-47.3, -28.4) | 6.2E-07 |
| DAG(16:1/18:3) | -38.5 (-45.9, -30.1) | 2.6E-10 |
| TAG52:3-FA16:1 | -38.3 (-44.9, -31.0) | 2.1E-13 |
| TAG54:5-FA22:4 | -38.3 (-47.0, -28.2) | 6.0E-07 |
| TAG50:5-FA18:3 | -38.3 (-46.8, -28.5) | 2.1E-07 |
| TAG51:3-FA18:1 | -38.2 (-44.8, -30.9) | 1.7E-13 |
| TAG50:2-FA16:0 | -38.2 (-46.0, -29.2) | 5.6E-09 |
| TAG51:2-FA16:0 | -38.1 (-45.5, -29.6) | 4.6E-10 |
| TAG52:1-FA16:1 | -37.9 (-46.4, -28.0) | 3.8E-07 |
| TAG52:1-FA20:1 | -37.8 (-46.8, -27.3) | 3.3E-06 |
| DAG(16:1/18:0) | -37.7 (-45.3, -29.0) | 2.8E-09 |
| TAG52:2-FA16:1 | -37.5 (-44.7, -29.2) | 2.9E-10 |
| TAG51:1-FA16:0 | -37.4 (-46.1, -27.4) | 9.4E-07 |
| TAG52:4-FA20:3 | -37.4 (-45.6, -28.0) | 1.0E-07 |
| TAG51:1-FA18:1 | -37.1 (-45.3, -27.7) | 1.1E-07 |
| TAG50:3-FA18:0 | -37.1 (-45.8, -27.0) | 1.6E-06 |
| TAG51:4-FA18:3 | -37.0 (-44.1, -29.1) | 7.8E-11 |
| TAG50:1-FA18:1 | -36.8 (-45.4, -26.8) | 1.2E-06 |
| TAG50:3-FA14:0 | -36.7 (-44.5, -27.9) | 1.1E-08 |
| TAG56:2-FA16:0 | -36.7 (-45.4, -26.5) | 2.3E-06 |
| TAG50:4-FA18:1 | -36.7 (-44.4, -27.9) | 1.0E-08 |
| TAG51:2-FA18:0 | -36.6 (-44.6, -27.5) | 5.0E-08 |
| TAG51:3-FA18:2 | -36.6 (-43.2, -29.2) | 1.9E-12 |
| TAG50:1-FA16:0 | -36.3 (-45.0, -26.3) | 2.0E-06 |
| TAG52:3-FA20:1 | -36.2 (-43.3, -28.3) | 1.3E-10 |
| PC(16:0/16:1) | -36.2 (-43.0, -28.5) | 3.5E-11 |
| TAG51:3-FA15:0 | -36.1 (-42.8, -28.6) | 9.0E-12 |
| TAG50:4-FA18:2 | -35.9 (-43.9, -26.7) | 1.3E-07 |
| TAG54:4-FA16:1 | -35.8 (-42.5, -28.2) | 2.0E-11 |
| TAG53:2-FA16:0 | -35.8 (-43.5, -26.9) | 2.8E-08 |
| TAG51:1-FA17:0 | -35.7 (-44.3, -25.9) | 1.8E-06 |
| TAG52:4-FA14:0 | -35.6 (-44.4, -25.5) | 5.1E-06 |
| TAG51:2-FA17:0 | -35.5 (-43.2, -26.6) | 4.1E-08 |
| TAG54:4-FA20:1 | -35.1 (-41.9, -27.7) | 2.5E-11 |
| TAG51:5-FA18:3 | -34.7 (-42.3, -26.2) | 2.0E-08 |
| DAG(16:0/18:1) | -34.7 (-42.1, -26.4) | 7.6E-09 |
| TAG54:6-FA22:5 | -34.7 (-43.1, -25.0) | 1.9E-06 |
| TAG51:4-FA18:1 | -34.4 (-41.4, -26.7) | 3.5E-10 |
| TAG52:4-FA16:1 | -34.0 (-40.8, -26.4) | 1.9E-10 |
| DAG(15:0/18:1) | -33.9 (-41.0, -26.0) | 2.1E-09 |
| TAG51:3-FA18:3 | -33.9 (-42.4, -24.2) | 3.8E-06 |
| TAG54:2-FA16:0 | -33.9 (-41.3, -25.5) | 1.9E-08 |
| TAG54:1-FA16:0 | -33.7 (-42.1, -24.2) | 2.5E-06 |
| TAG51:2-FA18:2 | -33.7 (-41.6, -24.8) | 2.8E-07 |
| TAG54:5-FA16:1 | -33.4 (-40.5, -25.4) | 4.3E-09 |
| TAG52:4-FA20:2 | -33.3 (-40.8, -24.7) | 8.9E-08 |
| TAG54:2-FA20:1 | -33.1 (-40.6, -24.7) | 5.9E-08 |
| TAG52:4-FA18:1 | -33.0 (-39.7, -25.6) | 2.0E-10 |
| TAG52:3-FA18:0 | -33.0 (-40.4, -24.6) | 4.6E-08 |
| TAG52:6-FA16:1 | -32.9 (-40.7, -24.1) | 3.8E-07 |
| TAG51:4-FA15:0 | -32.9 (-40.4, -24.4) | 9.0E-08 |
| TAG50:3-FA14:1 | -32.8 (-40.6, -24.0) | 3.6E-07 |
| TAG51:4-FA18:2 | -32.7 (-40.2, -24.3) | 6.5E-08 |
| DAG(16:1/20:2) | -32.7 (-38.8, -25.9) | 2.0E-12 |
| TAG53:2-FA18:1 | -32.7 (-39.7, -24.8) | 4.5E-09 |
| TAG52:5-FA18:1 | -32.5 (-39.5, -24.7) | 5.0E-09 |
| DAG(18:1/18:1) | -32.3 (-38.7, -25.1) | 6.8E-11 |
| TAG54:3-FA20:1 | -32.2 (-39.4, -24.2) | 1.6E-08 |
| TAG53:3-FA16:0 | -31.8 (-39.5, -23.1) | 6.0E-07 |
| TAG55:3-FA18:1 | -31.7 (-39.7, -22.6) | 3.0E-06 |
| TAG52:2-FA20:0 | -31.6 (-38.4, -24.0) | 3.8E-09 |
| TAG52:1-FA16:0 | -31.5 (-39.7, -22.2) | 6.8E-06 |
| TAG53:1-FA18:1 | -31.5 (-39.6, -22.2) | 6.4E-06 |
| TAG52:6-FA18:3 | -31.4 (-39.7, -22.1) | 9.6E-06 |
| TAG53:3-FA18:1 | -31.3 (-37.9, -24.0) | 6.2E-10 |
| DAG(18:0/18:1) | -31.1 (-38.4, -22.9) | 1.5E-07 |
| TAG52:1-FA18:1 | -31.1 (-38.9, -22.2) | 2.2E-06 |
| TAG53:2-FA17:0 | -31.0 (-38.0, -23.2) | 2.1E-08 |
| DAG(15:0/18:2) | -30.9 (-37.5, -23.6) | 1.0E-09 |
| TAG53:4-FA18:1 | -30.6 (-37.5, -23.0) | 1.0E-08 |
| TAG52:5-FA16:1 | -30.6 (-38.2, -22.0) | 1.1E-06 |
| TAG54:1-FA20:0 | -30.5 (-38.4, -21.6) | 4.5E-06 |
| CE(16:1) | -30.4 (-36.5, -23.7) | 3.6E-11 |
| CE(14:1) | -30.0 (-37.5, -21.5) | 1.4E-06 |
| TAG52:4-FA18:0 | -29.4 (-37.2, -20.6) | 7.8E-06 |
| TAG52:2-FA16:0 | -29.3 (-36.1, -21.8) | 3.0E-08 |
| DAG(16:0/18:2) | -29.3 (-37.1, -20.5) | 7.3E-06 |
| TAG54:3-FA16:0 | -29.3 (-36.9, -20.8) | 2.9E-06 |
| PC(14:0/20:3) | -28.9 (-35.0, -22.2) | 3.1E-10 |
| TAG52:4-FA22:1 | -28.8 (-36.0, -20.7) | 7.7E-07 |
| DAG(18:1/18:2) | -28.5 (-35.4, -20.9) | 1.1E-07 |
| TAG54:5-FA20:2 | -28.5 (-35.9, -20.3) | 2.2E-06 |
| TAG52:2-FA18:1 | -28.3 (-34.9, -21.1) | 1.8E-08 |
| TAG54:2-FA20:0 | -28.2 (-35.7, -19.8) | 5.3E-06 |
| PC(18:0/16:1) | -28.1 (-34.5, -21.1) | 5.7E-09 |
| DAG(18:1/20:1) | -28.1 (-34.1, -21.5) | 4.5E-10 |
| TAG54:3-FA18:1 | -28.0 (-35.6, -19.5) | 9.4E-06 |
| TAG52:3-FA22:1 | -27.8 (-34.7, -20.1) | 3.8E-07 |
| DAG(18:0/18:2) | -27.3 (-34.8, -19.0) | 9.7E-06 |
| TAG56:4-FA18:1 | -27.2 (-34.4, -19.2) | 3.1E-06 |
| TAG53:3-FA18:2 | -27.1 (-34.3, -19.1) | 3.8E-06 |
| TAG52:3-FA18:1 | -26.3 (-32.9, -19.1) | 2.4E-07 |
| LPC(16:1) | -26.3 (-30.9, -21.4) | 2.9E-16 |
| PC(14:0/18:2) | -26.3 (-31.9, -20.2) | 1.2E-10 |
| PC(14:0/18:1) | -26.3 (-32.2, -19.8) | 1.8E-09 |
| TAG53:3-FA17:0 | -26.1 (-33.3, -18.2) | 6.8E-06 |
| PC(15:0/18:2) | -25.9 (-30.7, -20.8) | 1.0E-14 |
| PC(18:1/16:1) | -25.0 (-30.0, -19.6) | 2.0E-12 |
| TAG52:3-FA16:0 | -24.6 (-31.5, -17.1) | 6.9E-06 |
| PE(18:0/20:3) | -24.4 (-30.0, -18.4) | 2.0E-09 |
| PC(16:0/20:3) | -24.2 (-29.5, -18.5) | 1.7E-10 |
| PE(18:1/20:3) | -24.0 (-29.6, -17.9) | 5.2E-09 |
| PC(18:2/20:3) | -22.8 (-28.4, -16.8) | 2.5E-08 |
| PC(18:1/20:3) | -22.7 (-27.7, -17.4) | 8.6E-11 |
| PC(15:0/18:1) | -22.5 (-27.1, -17.6) | 2.2E-12 |
| PI(18:0/20:3) | -22.0 (-27.2, -16.4) | 4.4E-09 |
| PC(18:2/16:1) | -21.9 (-26.2, -17.4) | 4.0E-14 |
| CE(15:0) | -21.8 (-27.0, -16.3) | 4.9E-09 |
| LPE(20:3) | -20.9 (-26.3, -15.2) | 8.5E-08 |
| PE(18:0/18:2) | -20.3 (-25.6, -14.5) | 2.6E-07 |
| LPE(16:0) | -19.9 (-24.7, -14.7) | 7.1E-09 |
| CER(14:0) | -18.8 (-23.3, -13.9) | 3.8E-09 |
| PE(18:0/18:1) | -17.6 (-22.7, -12.1) | 4.7E-06 |
| LPC(15:0) | -17.5 (-22.2, -12.6) | 1.3E-07 |
| SM(14:0) | -17.3 (-21.8, -12.6) | 4.1E-08 |
| LPE(18:0) | -16.4 (-20.8, -11.6) | 2.9E-07 |
| PC(16:0/18:2) | -15.6 (-19.3, -11.6) | 8.1E-10 |
| PC(16:0/18:1) | -15.5 (-20.1, -10.6) | 5.8E-06 |
| LPC(16:0) | -14.1 (-17.8, -10.2) | 4.1E-08 |
| SM(20:0) | -12.8 (-16.5, -9.1) | 3.4E-07 |
| TAG58:10-FA20:4 | 47.5 (29.9, 67.5) | 2.6E-06 |

1. Multivariable linear regression analysis was performed on log-transformed lipid species and adjusted for age, body mass index (BMI), alcohol consumption, and education.
2. Multiple hypothesis testing was corrected using Bonferroni correction. Statistical significance was defined as a Bonferroni-adjusted p-value <${10}^{-5}$.
3. Percentage difference was back-transformed linear regression coefficients, calculated as$100\times(e^{\beta}-1)$, with a 95% confidence interval. A negative percentage difference indicates that the lipid species levels are lower in non-Hispanic black women compared with non-Hispanic white women.

**Table S3. Number of Significant Lipid Species with ≥20% Absolute Percentage Difference between non-Hispanic Black (NHB) and non-Hispanic White (NHW) Women. ^a, b, c^**

| Super-pathway ^d^ | Sub-pathway | Super-pathway Lipid Species Count | Direction of Effect ^e^ | |
| --- | --- | --- | --- | --- |
|  |  |  | (↓) in NHB | (↑) in NHB |
| *Neutral Complex Lipids* | Triacylglycerols | 220 | 197 | 1 |
|  | Diacylglycerols |  | 19 | 0 |
|  | Cholesteryl Esters |  | 3 | 0 |
| *Phospholipids* | Phosphatidylcholines | 18 | 12 | 0 |
|  | Phosphatidylethanolamines |  | 3 | 0 |
|  | Lysophosphatidylcholines |  | 1 | 0 |
|  | Lysophosphatidylethanolamines |  | 1 | 0 |
|  | Phosphatidylinositols |  | 1 | 0 |

1. Multivariable linear regression analysis was performed on log-transformed lipid species and adjusted for age, body mass index (BMI), alcohol consumption, and education.
2. Multiple hypothesis testing was corrected using Bonferroni correction. Statistical significance was defined as a Bonferroni-adjusted p-value <${10}^{-5}$.
3. Percentage difference was back-transformed linear regression coefficients, calculated as$100\times\left( e^{\beta}-1 \right),$with a 95% confidence interval. Lipid species with an absolute percentage difference of 20% and above were listed.
4. Sphingolipids super-pathway did not contain any lipid species with a significant difference (≥20%) between groups and is therefore not included in this table.
5. Direction of effect indicated lower (↓) or higher (↑) lipid species in NHB compared with NHW. The number represented the lipid species count within each sub-pathway.

**Table S4. Lipid Sub-pathways Associated with Race (non-Hispanic Black vs non-Hispanic White) ^a, b^**

| Lipid Sub-pathway | Percentage Difference (%) ^c^ | Bonferroni Adjusted P-value |
| --- | --- | --- |
| Triacylglycerols | -30.1 (-37.0, -22.6) | 2.8E-10 |
| Diacylglycerols | -26.6 (-33.6, -18.7) | 5.2E-08 |
| Lysophosphatidylethanolamines | -15.8 (-20.5, -10.9) | 6.6E-08 |
| Phosphatidylcholines | -11.3 (-14.8, -7.6) | 2.3E-07 |

1. Multivariable linear regression analysis was performed on log-transformed lipid sub-pathways and adjusted for age, body mass index (BMI), alcohol consumption, and education.
2. Multiple hypothesis testing was corrected using Bonferroni correction. Statistical significance was defined as a Bonferroni-adjusted p-value <${10}^{-5}$.
3. Percentage difference was back-transformed linear regression coefficients, calculated as$100\times(e^{\beta}-1)$, with a 95% confidence interval. A negative percentage difference indicates that the lipid sub-pathway is lower in NHB women compared with NHW women.

**Table S5. Sub-pathways enriched with Lipid Species that Differ between Non-Hispanic White and Non-Hispanic Black Women, Bonferonni-adjusted P<**${10}^{-5}$.

| Lipid Pathway | Total Species | Significant Species | | Bonferroni Adjusted P-value |
| --- | --- | --- | --- | --- |
| *Neutral Complex Lipids* |  |  |  | |
| TAG | 518 | 198 | **2.09E-14** | |
| DAG | 58 | 19 | 0.30 | |
| CE | 26 | 3 | 0.99 | |
| *Phospholipids* |  |  |  | |
| PC | 61 | 14 | 0.89 | |
| PE | 72 | 4 | 1.00 | |
| LPC | 17 | 3 | 0.91 | |
| LPE | 10 | 3 | 0.59 | |
| PI | 10 | 1 | 0.97 | |
| *Sphingolipids* |  |  |  | |
| SM | 12 | 2 | 0.90 | |
| CER | 12 | 1 | 0.98 | |

Abbreviations: Triacylglycerols (TAG), Diacylglycerols (DAG), Cholesteryl Esters (CE), Phosphatidylcholines (PC), Phosphatidylethanolamines (PE), Lysophosphatidylcholines (LPC), Lysophosphatidylethanolamines (LPE), Phosphatidylinositols (PI), Sphingomyelins (SM), Ceramides (CER).

**Supplementary References**

1. Getz KR, Jeon MS, Luo C, Luo J, Toriola AT. Lipidome of mammographic breast density in premenopausal women. Breast Cancer Research. 2023;25(1):121.

2. Toriola AT, Appleton CM, Zong X, Luo J, Weilbaecher K, Tamimi RM, et al. Circulating Receptor Activator of Nuclear Factor-κB (RANK), RANK ligand (RANKL), and Mammographic Density in Premenopausal Women. Cancer Prev Res (Phila). 2018;11(12):789-96.

3. [Available from: <https://www.metabolon.com/>.

4. Evans AM, Bridgewater B, Liu Q, Mitchell M, Robinson R, Dai H, et al. High resolution mass spectrometry improves data quantity and quality as compared to unit mass resolution mass spectrometry in high-throughput profiling metabolomics. Metabolomics. 2014;4(2):1.

5. DeHaven CD, Evans AM, Dai H, Lawton KA. Organization of GC/MS and LC/MS metabolomics data into chemical libraries. Journal of cheminformatics. 2010;2(1):1-12.
